# 非小细胞肺癌*EGFR*突变非侵入性检测技术研究进展

**DOI:** 10.3779/j.issn.1009-3419.2018.12.09

**Published:** 2018-12-20

**Authors:** 世洋 袁, 叶青 邹, 军平 谢

**Affiliations:** 1 330006 南昌，南昌大学第二附属医院呼吸与危重症医学科 Department of Respiratory and Critical Care Medicine, the Second Affiliated Hospital of Nanchang University, Nanchang 330006, China; 2 330006 南昌，南昌大学第二附属医院江西省分子医学重点实验室 Jiangxi Province Key Laboratory of Molecular Medicine, the Second Affiliated Hospital of Nanchang University, Nanchang 330006, China

**Keywords:** 肺肿瘤, 表皮生长因子受体, 液体活检, 呼出气冷凝液, Lung neoplasms, Epidermal growth factor receptor, Liquid biopsy, Exhaled condensate

## Abstract

在过去的十年里，癌症患者的管理模式已经逐渐转向为基于分子突变检测的个体化模式。表皮生长因子受体（epidermal growth factor receptor, *EGFR*）基因突变是非小细胞肺癌（non-small cell lung cancer, NSCLC）的重要驱动因素，针对EGFR的靶向治疗和传统化疗相比，显示出显著的安全性和有效性。然而，并不是所有的*EGFR*突变患者都可接受EGFR靶向治疗，不同的突变类型往往预示着不同的临床结局，如敏感性突变*EGFR* 19-Del、L858R和耐药性突变20ins。此外，如今已经开发出第三代TKI药物Osimertinib（AZD9291）和Rociletinib（CO-1686）可使因*EGFR* T790M突变，导致初代TKI耐药的患者进一步获益。因此，治疗前了解患者*EGFR*突变状态，治疗过程中持续监测耐药基因*EGFR* T790M突变情况，对NSCLC患者靶向药物的管理有着重要的意义。最近几年来，“液体活检”技术得到快速的发展，让我们看到采用非侵入性方法以实时监测耐药性突变成为现实的可能。在本综述中，我们回顾了NSCLC中检测*EGFR*突变的多种非侵入性检测技术在不同液体样本的临床应用。

## Abstract (en)

Over the past decade, the management model of cancer patients has gradually shifted to individual mode based on molecular mutation detection. Epidermal growth factor receptor (*EGFR*) gene mutation is an important driving factor in non-small cell lung cancer (NSCLC). Compared with traditional chemotherapy, EGFR-targeted therapy shows significant safety and efficacy. However, not all patients with *EGFR* mutations are eligible for EGFR-targeted therapy, and different types of mutations often indicate different clinical outcomes, such as the sensitive mutations *EGFR* 19-Del, L858R, and the resistance mutation. In addition, the third-generation TKI drugs Osimertinib (AZD9291) and Rociletinib (CO-1686) have been developed to further benefit patients with primary TKI resistance caused by T790M mutation of *EGFR*. Therefore, detection of the *EGFR* mutation status of patients before treatment, and continuously monitoring the mutation of drug resistance genes during the treatment process is useful for the management of targeted drugs in NSCLC patients. In recent years, the rapid development of " liquid biopsy" technology has made it possible to use non-invasive methods to monitor drug resistance mutations in real time. In this paper, we reviewed the clinical application of various non-invasive detection techniques for *EGFR* mutations in NSCLC in different liquid samples.

表皮生长因子受体（epidermal growth factor receptor, *EGFR*）的突变是非小细胞肺癌（non-small cell lung cancer, NSCLC）的重要驱动因素，导致EGFR靶向治疗成为NSCLC的治疗选择^[1]^。虽然EGFR靶向治疗耐药的发生仍然不可避免，但发现其中约有60%是因*EGFR* T790M突变引起，这类患者可进一步获益于第三代TKI药物^[2, 3]^。因此，治疗前了解患者*EGFR*突变状态，治疗过程中持续监测耐药基因突变情况，对NSCLC患者靶向药物的管理有着重要的意义。为实时监测突变患者用药过程中耐药基因的突变情况，反复进行有创的方式采集组织样本，似乎不可取。而用于肿瘤取样的非侵入性方法以实时监测耐药性突变无疑是最佳的选择。近年来“液体活检”技术已得到快速的发展，除血液^[4]^、胸水^[5]^样本外，支气管肺泡灌洗液（bronchoalveolar lavage, BAL）^[6]^、呼出气冷凝液（exhaled breath condensate, EBC）^[7]^以及尿液^[8]^等也可用于*EGFR*突变的检测，并且同样显示了良好的灵敏度和特异度。在此，我们对上述几种液体样本材料的“液体活检”在检测NSCLC *EGFR*突变的现况和前景作一综述。

## 1 “液体活检”的生物学基础

癌细胞的快速更新导致肿瘤衍生的核酸和囊泡不断地释放至血液系统中。并且活的肿瘤细胞也可以从原发灶中脱落至血流中，这类细胞称之为循环肿瘤细胞（circulating tumour cells, CTCs）。血液系统中可以包含来自于肿瘤组织和其他组织分泌或脱落的细胞、囊泡（如外泌体）、细胞游离DNA（cell-free DNA, cfDNA）和RNA（cfRNA）以及蛋白质等物质。“液体活检”就是检测血液中的具有原发肿瘤组织遗传信息的循环肿瘤DNA（circulating tumour DNA, ctDNA）、肿瘤衍生的RNA（主要为microRNA）、外泌体以及CTCs等。与侵入性检测方法相比，临床医生通过“液体活检”这一非侵入性检测方法实时的了解患者体内癌症的进展情况显得更为便捷^[9]^。

## 2 “液体活检”的多种检测平台的比较

“液体活检”标本的分析是具有挑战性的，因为循环cfDNA主要由正常细胞的DNA和癌症患者中存在的相对小且高度可变的ctDNA部分组成。而传统DNA分析方法（如Sanger测序）的灵敏度不足以检测癌症患者血浆ctDNA中的体细胞突变。因此，开发更灵敏的测序分析在NSCLC的治疗中尤为重要。目前，已有多种半定量和定量基因分析平台用于血浆基因分型，以下将简单介绍这些检测平台之间的差异。

基于PCR的多种半定量基因检测平台包括扩增难治性突变系统（amplified refractory mutation system, ARMS）^[10]^、Cobas^[4]^和肽核酸（peptide nucleic acid, PNA）钳^[11]^等可达0.1%的检测下限，并且在多项研究中显示具有较高的特异性和适度的敏感性。数字PCR（digital polymerase chain reaction, dPCR）作为DNA定量的新技术，实现了单分子DNA绝对定量。微滴数字PCR（droplet digital PCR, ddPCR）^[12]^和磁珠乳液扩增（Bead, Emulsion, Amplification, Magnetic, BEAMing）^[13]^技术是基于dPCR技术的新的基因突变检测平台，检测更为精细，下限分别可达0.001%和0.01%。利用这些高灵敏度平台进行的血浆基因分型检测已被证明可快速检测和定量转移性NSCLC患者血浆中存在的突变型cfDNA水平，具有高度特异性。上述这些检测平台主要优势在于快速、高灵敏度、不需生物信息学分析以及具有成本效应，便于临床推广。而不足之处在于：只能监测已知的突变。

此外，非靶向全基因组分析能够在不事先知道肿瘤中可能存在的畸变的情况下鉴定肿瘤特异性改变。因此，可以利用这些方法从源头发现治疗耐药的基因变化，并鉴定癌症患者中新的可操作靶标。下一代测序（next-generation gene sequencing, NGS）是一种技术，涉及将DNA片段固定在固体载体上并读取序列作为DNA合成过程的一部分。使用NGS，可以在单个反应中产生数百万个ctDNA序列，随后进行比对并与参考基因组或从同一患者（非恶性组织，通常是外周血单核细胞）获得的种系DNA进行比较，使得可以鉴定相对于参考序列的核苷酸变化^[14]^。现在已经设计了几种基于NGS的方法，不仅能够检测点突变和插入、缺失或重排，还能够检测拷贝数改变和基因融合^[15]^。NGS的优势在于探索治疗抗性新的突变机制，主要推广难点在于检测周期长，需要生物信息学分析，并且检测昂贵。

## 3 “液体活检”在NSCLC患者*EGFR*突变检测中的临床应用

### 3.1 基于血液的“液体活检”

对患者血液中ctDNA进行分子检测是目前应用最为广泛和成熟的“液体活检”。Sacher等^[16]^的前瞻验证性研究纳入120例新诊断和60例初代EGFR-TKI耐药的非鳞癌NSCLC患者，采用ddPCR法检测所有患者的血浆cfDNA的*EGFR* 19-del、L858R以及T790M突变状态，并把患者组织基因型作为参考标准，比较其检测的敏感度和特异度。研究结果显示，所检测EGFR 3个位点的敏感度大致相似，约70%-80%，但随患者肿瘤转移部位的增多、合并肝或骨转移而提高，认为血浆*EGFR*突变的检测敏感性疾病负荷以及肝或骨转移相关，这可能提示肿瘤的ctDNA增加；检测19-del、L858R的特异度高达100%（95%CI: 97%-100%）；检测T790M特异度相对较低，仅有63%，即有8例血浆中检测出T790M阳性，匹配的组织基因型为T790M阴性，但接受第三代EGFR-TKI治疗有效，被认为这是由肿瘤组织存在的异质性引起。基于其他平台如BEAMing^[17]^、Cobas^[4]^、NGS^[18]^等检测血液cfDNA也显示出相似的结果：检测血浆*EGFR*敏感突变具有显著的特异性，可用于筛选阳性人群而避免再次侵入性活检；同时也可检测出因肿瘤异质性在组织基因分型中遗漏的T790M阳性突变患者；但敏感度相对欠佳，存在较大的假阴性（30%）可能，尚需要肿瘤组织活检。

CTCs可以从癌症患者的血液中分离，其研究前景广泛，不仅可以充当作为癌症预后以及判断治疗疗效的生物标志物，也可以用分离的CTCs进行分子检测明确原发肿瘤的遗传特性指导和检测靶向药物疗效，未来更可以通过建立CTC衍生的异种移植物（CTC derived xenografts, CDXs）模型探讨多耐药的癌症细胞接受新药物的治疗效果^[19-21]^。然而，血液中的CTCs丰度较低，每毫升血液存在有限个数的CTCs，对开发进一步的临床应用尚需要更有效的富集技术及更高精度的分子检测方法^[22]^。近年来，CTCs有效的富集技术已得到较大的发展，有数篇综述对此进行了全面而系统的评价^[23, 24]^，此处我们着重探讨利用CTCs进行分子检测的可行性。Gao等^[25]^通过建立组合免疫磁珠（EpCAM, MUC1, EGFR）富集肺癌细胞，采用ddPCR芯片技术检测CTCs的*EGFR* L858R突变状态。结果显示，富集有效率高达90%以上；在L858R突变的H1975细胞中有检测到*EGFR* L858R突变和*EGFR*野生型突变，在没有L858R突变的A549细胞中仅检测到*EGFR*野生型突变；在已知的2例*EGFR* L858R突变的患者中检测到结果与组织基因突变型一致，而在已知没有*EGFR*突变的1例肺癌患者以及2名健康人中检测到*EGFR*野生型突变。因此，作者认为采用ddPCR芯片技术检测CTCs的*EGFR*突变状态用于指导临床用药是具有可行性的，但仍需要更大样本量临床研究验证。

外泌体在细胞间交换分子信息方面具有重要作用，它们已被证明含有蛋白质以及一系列核酸，包括DNA、mRNA和miRNA等^[26]^。因此，肿瘤细胞分泌的外泌体可携带其本身的遗传信息，检测肿瘤患者体液的外泌体DNA或RNA可用于反映肿瘤组织的遗传特性。Krug等^[27]^从参加TIGER-X（NCT01526928）临床研究的患者筛选出84例患者，收集患者相匹配的肿瘤组织及血液样本，分离提取血液中的外泌体的DNA和RNA（合称exoNA）以及血液中的ctDNA，比较EXO1000 NGS平台检测exoNA与EBMAing平台检测ctDNA *EGFR*突变状态的敏感度和特异度。结果显示，与肿瘤组织*EGFR*突变型作为参考标准，exoNA检测患者*EGFR*敏感突变型和T790M突变的敏感度均高于ctDNA，差异有统计学意义（分别为*P*=0.004和*P*=0.003），突变拷贝数和突变等位基因分数（mutant allele fraction, MAF）exoNA显著高于ctDNA可能是这一差异的原因之一。在循环中核酸水平低的情况下，例如肿瘤负荷低，胸内疾病或早期发现癌症的患者，基于exoNA的液体活检平台可能特别有益。Castellanos-Rizaldos等^[28]^的研究也显示了相似的结果，其设计敏感等位基因特异性qPCR来检测分离提取的exoNA *EGFR* T790M突变状态。结果显示，使用肿瘤活检结果作为参考标准时，检测exoNA上的T790M突变达到了92%的灵敏度和89%的特异度。对胸内疾病（M0/M1a）患者获得了较高灵敏度（88%），因此认为对于这些患者，基于exoNA的液体活检检测更有优势。总之，相对ctDNA，exoNA显示出更高的突变拷贝数和突变等位基因分数，这使得基于exoNA的液体活检可获得更高的灵敏度。

### 3.2 基于胸腔积液的“液体活检”

目前临床上针对胸腔积液的检测，仅限于常规、生化以及胸水细胞学和染色体等，诊断水平停留在“找见肿瘤细胞”或“未找见肿瘤细胞”。而早在2006年，Cavalli等^[29]^认为在胸腔积液中体细胞*EGFR*突变的研究是切实可行的，在许多NSCLC患者中，胸腔积液采样被视为肿瘤细胞采集的更具侵入性方法的替代方案。该研究共纳入了68例一线化疗失败的合并有恶性胸腔积液的NSCLC患者。检测所有患者胸腔积液细胞组织蜡块DNA的*EGFR* 19-Del和L858R突变状态，41例阳性患者采用吉非替尼治疗（实验组），27例阴性患者采用铂类为基础的二线化疗（对照组）。结果显示，实验组的无进展生存期（progression-free survival, PFS）、客观反应率（objective response rate, ORR）、疾病控制率（disease control rate, DCR）均优于对照组。认为在晚期非小细胞肺癌合并恶性胸腔积液患者中检测*EGFR*突变是可行的且检出率高[60.3%（41/68）]，靶向治疗是这些突变患者安全有效的方法。Yeo等^[30]^的研究纳入了37例患有恶性胸腔积液的NSCLC患者，通过PNA钳和直接测序法检测患者肿瘤组织、胸腔积液和血清的*EGFR*突变状态。结果显示，与肿瘤组织相比，胸腔积液*EGFR*突变检测敏感性为89%、特异性为100%。他们认为，和血液样本相比，胸腔积液的检测效能和肿瘤组织相当，能够更敏感、更准确地检测*EGFR*突变状态。

### 3.3 基于支气管肺泡灌洗液的“液体活检”

随着呼吸内镜技术的快速发展，支气管冲洗、刷洗或支气管肺泡灌洗技术已成为常规呼吸内镜技术，获得的BAL（在此我们把支气管冲洗液或刷洗液也称之为BAL）通常可用于疾病的诊断。Kawahara等^[31]^为探索在BAL样本上清cfDNA中检测*EGFR*突变的可行性，研究共纳入74例含有BAL样本的肺腺癌患者，已知的肿瘤组织病理标本*EGFR*突变状态作为参考标准，采用PCR技术分析BAL样本上清cfDNA *EGFR*突变的敏感度为88.0%，特异度为100%。Park等^[32]^也做了类似的研究，通过PNA钳技术检测BAL样本分离的cfDNA *EGFR*突变状态，结果显示BAL上清的cfDNA与肿瘤组织检测出*EGFR*突变的结果一致率高达91.7%，具有较高的诊断价值，也证实了在BAL样本检测*EGFR*突变的可行性。目前这一结论尚缺乏大样本、多中心的临床研究验证，而临床支气管内镜诊断肺癌的发展方向是如何更加精准地获取肿瘤组织标本，如支气管超声引导的针吸活检术（endobronchial ultrasound-transbronchial needle aspiration, EBUS-TBNA）^[33]^。

### 3.4 基于呼出气冷凝液的“液体活检”

呼出气体冷凝液（exhaled breath condensate, EBC）是呼出气体的液体形式。它是一种非侵入性器官特异性无细胞生物液，来自气道衬里液，已知是肺癌特异性DNA的来源。十年前，Paradiso等^[34]^在23例NSCLC患者中检测到1例*EGFR* 19-DEL突变，并且在EBC和肿瘤组织中检测到的基因型不一致。因此认为，在EBC中检测体细胞*EGFR*基因突变状态是不可行的。而Zhang等^[35]^在重度吸烟的肺鳞癌患者的EBC中检测到EGFR 19-Del，随后通过纤支镜获取标本组织并EGFR检测提示确实存在19-Del突变，并在使用吉非替尼治疗后显示出良好的疗效。他们认为能检测到*EGFR*突变可能归因于PCR方法的敏感性或肿瘤位置。最近，Smyth等^[36]^的研究中共纳入了19例具有*EGFR*阳性突变的晚期NSCLC患者，其中10例为T790M突变，9例为*EGFR*敏感突变。收集患者相应靶向治疗前匹配的血液和EBC样本，用于检测T790M突变。结果显示，在10例T790M阳性患者的EBC标本中检测出9例T790M阳性，而血液标本检出7例；在9例*EGFR*敏感突变患者的EBC和血液标本均为检测T790M阴性突变。其原因可能是，EBC样本比血液样本含有更低水平的野生型DNA及内源性核酸酶活性，因此更适用于肺部疾病的检测。但研究样本量较少，不足以进行统计分析，需要进一步研究验证EBC作为*EGFR*突变肺癌患者血浆中检测EGFR-T790M的替代或辅助的作用。

### 3.5 基于尿液的“液体活检”

由于cfDNA或ctDNA片段小、分子量低，可以经肾过滤后从尿液排出。因此，尿液可能是研究ctDNA的有价值的来源^[37]^。研究^[38, 39]^表明，在血浆ctDNA中检测到的肿瘤特异性遗传改变（如点突变和甲基化谱等），也可以在匹配的尿ctDNA中检测。Reckamp等^[40]^的研究共纳入63例患者，采用突变富集PCR联合NGS检测尿液和血液ctDNA *EGFR* 19-Del、L858R和T790M突变状况。结果显示：使用肿瘤组织检测结果作为参考标准，尿液中（所有样本，尿量为10 mL-100 mL）^[38, 39]^突变检测的敏感性：T790M为72%，L858R为75%，19-Del为67%。而对于推荐尿量为90 mL-100 mL的样本，EGFR突变检测灵敏度：T790M为93%，L858R为80%，19-Del为83%。使用从健康供体和非NSCLC转移性癌症人群获得的尿样测定EGFR尿液测定的特异性：T790M为96%，L858R为100%，19-Del为94%。检测血浆的灵敏度：T790M为93%，L858R为100%，19-Del为87%。使用从健康供体和患有非NSCLC转移性癌症的人群获得的血浆样品确定EGFR血浆测试的特异度：T790M为94%，L858R为100%，19-Del为96%。值得注意的是，尿液和血浆测试共同确定了另外12例T790M阳性病例，这些病理组织标本检测为T790M阴性，接受rociletinib治疗期间监测到有9例患者在第21天观察到尿T790M水平的快速降低。因此认为尿液可以作为EGFR检测的非侵入性来源样本，和血液标本相比，具有类似的高灵敏度和特异度。Chen等^[8]^招募了150例*EGFR*敏感突变并接受EGFR-TKIs的NSCLC患者参与系列的检测研究，在TKI治疗开始前，使用ddPCR法对患者原发肿瘤组织样本、血液和尿液样本进行*EGFR*突变检测，随后每间隔1个月采集患者血液和尿液标本用于*EGFR*突变检测，共持续9个月。结果显示，治疗前尿ctDNA *EGFR*突变检出结果与肿瘤组织结果一致率达88%，且几乎和血液标本检出结果一致。采用尿液监测期间，53%的患者出现T790M突变，这些患者在血浆ctDNA中也得到证实。认为，尿液cfDNA可能是常规基于原发组织的*EGFR*突变检测的潜在替代方案。尿液*EGFR*突变检测灵敏度与血浆结果相当，具有用于监测EGFR-TKI治疗的临床效用。目前，定量检测尿液ctDNA *EGFR*突变状态仍然是具有挑战性的，主要因存在的ctDNA丰度低。另外，尿液ctDNA含量也可能受到患者临床状态的影响^[41]^。未来随着DNA扩增和测序技术的进一步发展，相信尿液活检作为真正意义上的非侵入性替代方案会得到更大的发展。

## 4 总结与展望

搭载不同检测平台的多种液体样本的“液体活检”作为非侵入性技术实时监测*EGFR*突变已显示其优越性和临床应用前景。基于胸腔积液的“液体活检”应当是目前首选检测*EGFR*突变的组织替代方案，不仅在于其所含ctDNA丰度高，而且可提供肿瘤的细胞学材料。血液样本是目前应用最为广泛的“液体活检”材料，其临床价值显著，但血液循环中极低丰度的ctDNA，对检测技术带来了挑战，最直观的体现在于灵敏度不高。尿液样本中ctDNA和血液样本同源且片段更为细小，ctDNA的含量容易受患者临床状况所影响，给检测带来相当的难度，但可通过增加尿液量收集ctDNA达到和血液标本几乎一致的检测效能，且尿液是真正意义上的非侵入性材料，值得进一步开发利用。我们认为，检测外泌体DNA或RNA是最值得开发的项目，因为exoDA中的突变拷贝数和MAF分数比ctDNA更高，体现出的检测效能也更高，不足的是操作步骤较复杂且需要搭载灵敏度更高的检测平台。目前这些材料体现出检测NSCLC *EGFR*突变临床应用的潜能，除血液和胸腔积液，其他液体材料仍缺乏相应的临床研究结果证实，相信未来将会被一一证实，尤其是应用尿液外泌体检测肿瘤的分子状态。
